# Supplementary material for: Cell-cycle-resolved Hi-C reveals unexpected plasticity of A/B compartments across interphase
Source: eLife. 2026 Oct 6;15:RP110073. doi: 10.7554/eLife.110073 (PMC13641764; doi:10.7554/eLife.110073)
Supplement: Peer review history

# Editor report: eLife Assessment

**DOI:** 10.7554/eLife.110073.3.sa0

**Authors:** Melike Lakadamyali

**Affiliations:** University of Pennsylvania United States

**Keywords:** Solid, Valuable

This article provides **valuable** insight into how genome organization changes as cells progress through the cell cycle after mitotic exit, identifying two sharp genome remodeling events at G1-S and to a lesser extent, at S-G2 transitions. The conclusions are supported by **solid**, rigorous data, including sequencing and orthogonal imaging data. The use of sorted unsynchronized cells rather than cells treated with drugs is a particular strength.

# Referee report: Reviewer #1 (Public review)

**DOI:** 10.7554/eLife.110073.3.sa1

This work convincingly shows that, rather than gradually "evolving" throughout interphase, global chromatin architecture undergoes unexpectedly sharp remodeling at G1-S (and to a lesser extent, S-G2) transitions. By applying "standard" Hi-C analyses on carefully sorted cells, the authors provide an excellent temporal view of how global chromatin architecture is changed throughout the cell cycle. They show a surprisingly abrupt increase in compartmentation strength (particularly interactions between the "active" A compartments) at G1-S transition, which is slightly weakened at S-G2 transition. Follow-up experiments show convincingly that the compartment "maturation" does not require the DNA synthesis accompanying S phase per se, but the authors have not identified the responsible factors (work for future publications). The possible biological ramifications of these architectural changes (setting up potential replication "factories", and/or facilitating transcription-replication conflict resolution, both more pertinent for the active A compartments, which are most affected) have been well discussed in the article, but still remain speculative at this stage.

My major criticism of this article is aimed more at the state of the field in general, rather than this specific article, but it should be discussed to give a more balanced view: what actually is a chromatin compartment? Chromosomal tracing and live tracking experiments have shown that the majority of "structures" identified from Hi-C experiments are statistical phenomena, with even "strong" interactions only being infrequent and transient. A-B compartments are "built up" from multiple very low-frequency "interactions", so ascribing causal effects for genome functions is even tougher. As a result, I have very little confidence in the results of the authors' polymer simulations and their inferred "peninsula" A compartment structures without any other supporting experimental data.

Comments on revised version.

The authors have included orthogonal DNA FISH evidence to support their claims which greatly strengthens the manuscript. Their further precisions within the discussion have answered all of my previous concerns with the manuscript.

# Referee report: Reviewer #2 (Public review)

**DOI:** 10.7554/eLife.110073.3.sa2

Summary:

This manuscript by Choubani et al presents a technically strong analysis of A/B compartment dynamics across interphase using cell-cycle-resolved Hi-C. By combining the elegant Fucci-based staging system with in situ Hi-C, the authors achieve unusually fine temporal resolution across G1, S, and G2, particularly within the short G1 phase of mESCs. The central finding that A/B compartment strength increases abruptly at the G1/S transition, stabilizes during S phase, and subsequently weakens toward G2 challenges the prevailing view that compartmentalization strengthens monotonically throughout interphase. The authors further propose that this "compartment maturation" is triggered by S-phase entry but occurs independently of active DNA synthesis, and that it involves a consolidation and large-scale reorganization of A-compartment domains.

Strengths:

Overall, this is a thoughtfully executed study that will be of broad interest to the 3D genome community. The data are of high quality, and the analyses are extensive, albeit not completely novel. In particular, previous work (Nagano et al 2017 and Zhang et al 2019) has shown that compartments are re-established after mitosis and strengthened during early interphase, and single-cell Hi-C studies have reported changes in compartment association across S phase. In particular, Nagano et al show that DNA replication correlates with a build-up of compartments, similar to what is presented here, with the authors' conclusion that compartment strength peaks in early S. The idea that it weakens toward G2, rather than continuing to strengthen, appears to be novel and differs from the prevailing framing in the literature.

Comments on revised version.

The authors have responded constructively to my major conceptual concerns. The distinction between DNA synthesis and replication initiation has been clarified appropriately. The additional insulation analysis substantially strengthens the argument that compartment maturation is not simply a consequence of changing loop extrusion dynamics, although I would encourage slightly more cautious wording regarding "independence" from cohesin-mediated extrusion. The peninsula model is now framed appropriately as a heuristic interpretation and supported by orthogonal imaging data. Finally, the discussion of conservation across cell types has been appropriately tempered. Overall, I believe the manuscript has been significantly improved.

# Author comment: Author response

**DOI:** 10.7554/eLife.110073.3.sa3

**Authors:** Linda Choubani, Hisashi Miura, Takako Ichinose, Asami Oji, Saori Takahashi, Rory T Cerbus, Ichiro Hiratani

**Affiliations:** RIKEN Center for Biosystems Dynamics Research Kobe Japan; RIKEN Center for Biosystems Dynamics Research Kobe Japan; RIKEN Center for Biosystems Dynamics Research Kobe Japan; The University of Tokyo Tokyo Japan; RIKEN Center for Biosystems Dynamics Research Kobe Japan; RIKEN Center for Biosystems Dynamics Research Kobe Japan; RIKEN Center for Biosystems Dynamics Research Kobe Japan

The following is the authors’ response to the original reviews.

**Public Reviews:**

**Reviewer #1 (Public review):**

This work convincingly shows that, rather than gradually "evolving" throughout interphase, global chromatin architecture undergoes unexpectedly sharp remodeling at G1-S (and to a lesser extent, S-G2) transitions. By applying "standard" Hi-C analyses on carefully sorted cells, the authors provide an excellent temporal view of how global chromatin architecture is changed throughout the cell cycle. They show a surprisingly abrupt increase in compartmentation strength (particularly interactions between the "active" A compartments) at G1-S transition, which is slightly weakened at S-G2 transition. Follow-up experiments show convincingly that the compartment "maturation" does not require the DNA synthesis accompanying S phase per se, but the authors have not identified the responsible factors (work for future publications). The possible biological ramifications of these architectural changes (setting up potential replication "factories", and/or facilitating transcription-replication conflict resolution, both more pertinent for the active A compartments, which are most affected) have been well discussed in the article, but still remain speculative at this stage.

We thank Reviewer #1 for their positive and constructive assessment of our work, and we agree that the questions of responsible factors and biological ramifications are important directions for future studies.

My major criticism of this article is aimed more at the state of the field in general, rather than this specific article, but it should be discussed to give a more balanced view: what actually is a chromatin compartment? Chromosomal tracing and live tracking experiments have shown that the majority of "structures" identified from Hi-C experiments are statistical phenomena, with even "strong" interactions only being infrequent and transient. A-B compartments are "built up" from multiple very low-frequency "interactions", so ascribing causal effects for genome functions is even tougher. As a result, I have very little confidence in the results of the authors' polymer simulations and their inferred "peninsula" A compartment structures without any other supporting experimental data.

We thank the reviewer for raising this important conceptual point. This issue extends beyond the scope of the present study but reflects an important ongoing discussion in the 3D genome field regarding the biological interpretation of chromatin compartments.

We agree that Hi-C interactions should not be interpreted as stable pairwise contacts present in every cell. A growing body of evidence from chromatin tracing and live-cell imaging studies has demonstrated that many chromatin interactions identified by Hi-C are probabilistic and dynamic, with substantial cell-to-cell variability. Relatively speaking, however, A/B compartment organization represents a robust population-level property of genome organization that is highly reproducible across biological replicates and closely correlates with multiple independent genomic features. In particular, replication timing (RT) correlates very well with A/B compartment organization, with early and late RT domains corresponding to A and B compartment domains, respectively.

Furthermore, single-cell DNA replication sequencing (scRepli-seq) analyses have revealed remarkably low cell-to-cell variability in RT, suggesting that RT profiles and A/B compartment organization reflect biologically meaningful and relatively stable features of nuclear architecture rather than purely statistical artifacts. Thus, while individual chromatin contacts may be transient and probabilistic, the megabase-scale compartment organization inferred from them appears sufficiently reproducible to support reproducible RT programs and other genome functions. Additional support comes from decades of work on DNA replication demonstrating that spatiotemporal replication patterns, visualized as replication foci following short EdU pulses, are remarkably reproducible between individual cells throughout S-phase progression. These patterns reveal clear spatial segregation between early-replicating A-compartment regions and late-replicating B-compartment regions even at the single-cell level.

To directly address the reviewer’s concern that A/B compartment organization might represent only an ensemble-level statistical phenomenon without biological relevance at the single-cell level, we performed L1/B1-EdU DNA FISH on asynchronous mESCs and MC12 embryonic carcinoma cells. L1 elements are enriched in B compartment domains, while B1 elements are enriched in A compartment domains, allowing visualization of compartment segregation in individual nuclei across the cell cycle. This single-cell analysis confirmed our Hi-C findings: compartment segregation increased from G1 to early S, remained elevated throughout S phase with reduced cell-to-cell variability, and then weakened in G2. Thus, compartment segregation is detectable in single cells, and the temporal dynamics of compartment maturation identified by population Hi-C were independently recapitulated at single-cell resolution. We have added a new Results section describing these findings titled “Stepwise A/B compartment reorganization during interphase is conserved at single-cell resolution”, including new Figure panels 2D–H and Figure S5.

Regarding the polymer simulations, we agree that these models should be interpreted with caution. We do not view them as direct representations of individual nuclei, but rather as heuristic models that help visualize structural trends present in the Hi-C data. To make this point explicit, we have added the following statement to the revised manuscript: “We note that these models are derived from population-averaged Hi-C data and should therefore be interpreted as a heuristic framework for understanding A/B compartment dynamics, rather than as definitive representations of individual nuclei.”

That said, we did try to provide orthogonal experimental support for the "A peninsula" model by performing DNA FISH. In brief, we measured distances between probe pairs spanning two A domains on chromosomes 2 and 15 across different cell-cycle stages. We observed significant increases in inter-probe distances from G1 to early/mid S, with the most pronounced changes involving the central probes (i.e., probes located near the domain center), consistent with physical extension of the A domain during S phase. While these data do not prove the exact geometry depicted by the model, these findings provide independent experimental support for the peninsula model as a simplified but biologically grounded interpretation of the Hi-C data. These results are described in the Results section titled “A-compartment consolidation during S-phase involves enhanced long-range contacts and structural reorganization” and are presented in new Figure panels 5D–F and Figure S12.

We thank the reviewer again for raising this important conceptual issue, which prompted us to better clarify both the biological interpretation and the limitations of our analyses.

Specific minor points:

(1) A better explanation for how Figure 1E was generated is required, because this figure could be very misleading. Figure 1F and all other cis-decay plots (and the Hi-C maps themselves) show that the strongest interactions are always at smaller genomic separations, so why should there be more "heat" at the megabase ranges in Figure 1E?

We appreciate the reviewer's observation. The apparent discrepancy is simply due to the fact that the decay plot (Fig. 1E in the original submission, now Fig. S2C) does not include the shortest-range interactions. The lowest distance plotted is 25 kb, following the method originally described in Nagano et al. (Nature, 2017), which we used as a reference. The shortest-range interactions (below 25 kb) are indeed the most enriched, as seen on the diagonal of the Hi-C maps (Fig. 2A) and in the standard cis-decay plot (Fig. 1F in the original submission, now Fig. S2F). With the 25 kb cutoff in place, the "heat" observed at megabase distances (specifically 12–50 Mb) in early/mid G1 corresponds to the dark, non‑specific band around the diagonal visible in the Hi-C maps at the same time points. This is also reflected in the cis-decay plot (Fig. S2F), where distances in that range appear above the expected curve (a "bump" rather than a linear decay).

To avoid confusion, we have updated the figure legend accordingly (Fig. S2C): “(C) Contact decay profiles for all cell cycle phases, plotted from 25 kb to 50 Mb, illustrating a continuum of cis-interactions and a progressive shift from long-range (> 12 Mb) to short-range (< 1 Mb) interactions during the G1-to-S phase transition.”

We hope this explanation clarifies the figure.

(2) An ultra-high-resolution Hi-C study (Harris et al., Nat Commun, 2023) identified very small A and B compartments, including distinctions between gene promoters and gene bodies, raising further questions as to what the nature of a compartment really is beyond a statistical phenomenon. It is unreasonable to expect the authors to generate maps as deep as this prior study, but how much do their conclusions change according to the resolution of their compartment calling? The authors should include a balanced discussion on the "meaning" of A/B compartments.

We thank the reviewer for highlighting recent ultra-high-resolution work, such as Harris et al. (Nat Commun, 2023), which reveals compartment-like features at much finer genomic scales. We agree that these findings raise important questions regarding the scale-dependence and interpretation of A/B compartmentalization.

In our study, we specifically focus on coarse-grained compartment organization, analyzed across multiple resolutions (from ~1 Mb to sub‑megabase scales). Importantly, the key conclusions, including the abrupt strengthening of compartmentalization at the G1/S transition, are robust across these resolutions.

We also note that fine-scale compartment-like features likely operate under different rules than larger-scale compartments. Recent evidence suggests that these "micro‑compartments" are more dynamic and transient (Harris et al., Nat Commun, 2023; Goel et al., Nat Struct Mol Biol, 2025), whereas the large-scale compartments analyzed here capture more stable, global segregation patterns. Understanding how these two regimes relate to one another remains an important open question.

We have added the following statement in the Discussion acknowledging the scale-dependent nature of compartmentalization: “At the same time, recent ultra-high-resolution Hi-C studies [36,37] have revealed compartment-like features at much finer genomic scales, emphasizing that A/B compartmentalization is, to some extent, inherently scale-dependent. Understanding how these fine-scale, often transient micro-compartments relate to the more stable, large-scale segregation patterns described here will be an important direction for future studies.”

**Reviewer #2 (Public review):**

Summary:

This manuscript by Choubani et al presents a technically strong analysis of A/B compartment dynamics across interphase using cell-cycle-resolved Hi-C. By combining the elegant Fucci-based staging system with in situ Hi-C, the authors achieve unusually fine temporal resolution across G1, S, and G2, particularly within the short G1 phase of mESCs. The central finding that A/B compartment strength increases abruptly at the G1/S transition, stabilizes during S phase, and subsequently weakens toward G2 challenges the prevailing view that compartmentalization strengthens monotonically throughout interphase. The authors further propose that this "compartment maturation" is triggered by S-phase entry but occurs independently of active DNA synthesis, and that it involves a consolidation and large-scale reorganization of A-compartment domains.

Strengths:

Overall, this is a thoughtfully executed study that will be of broad interest to the 3D genome community. The data are of high quality, and the analyses are extensive, albeit not completely novel. In particular, previous work (Nagano et al 2017 and Zhang et al 2019) has shown that compartments are re-established after mitosis and strengthened during early interphase, and single-cell Hi-C studies have reported changes in compartment association across S phase. In particular, Nagano et al show that DNA replication correlates with a build-up of compartments, similar to what is presented here, with the authors' conclusion that compartment strength peaks in early S. The idea that it weakens toward G2, rather than continuing to strengthen, appears to be novel and differs from the prevailing framing in the literature.

We thank Reviewer #2 for their thoughtful assessment and critique. We address their specific concerns below.

Weaknesses:

That said, several aspects of the conceptual framing and interpretation would also benefit from further clarification, and the mechanistic interpretation of the reported compartment dynamics requires more careful positioning relative to established models of genome organization. Specific concerns are outlined below:

(1) One of the major conclusions of the study is that compartment maturation does not require ongoing DNA replication. However, the interpretation would benefit from more precise wording. Thymidine arrest still permits licensing, replisome assembly, and other S-phase-associated chromatin changes upstream of bulk DNA synthesis. Therefore, their data, as presented, demonstrate independence from DNA synthesis per se, but not necessarily from the broader replication program. Please clarify this distinction in the text and interpretations throughout the manuscript.

We thank the reviewer for this important distinction. We agree with their point and have never claimed that compartment maturation is independent of the broader replication program. That is why we carefully used the term "active DNA synthesis" rather than "replication" throughout the manuscript.

However, we acknowledge that one sentence in the text was ambiguous. The original sentence read: “These results confirm that the cell population was successfully synchronized at the G1/S boundary, representing a pre-replicative state where replication had not yet initiated, although cell-cycle markers indicated entry into S-phase.”

We have now revised it to: “These results confirm that the cell population was successfully synchronized at the G1/S boundary, representing a state where the replication program (including origin licensing, replisome assembly, and helicase activation) has been initiated, as indicated by cell-cycle markers, but ongoing DNA synthesis (elongation) is blocked. ”

This clarifies that compartment maturation is independent of active DNA synthesis (elongation) but not necessarily independent of upstream replication-associated processes. The change has been made in the manuscript.

(2) A major conceptual issue that is not addressed at all is the well-established anti-correlation between cohesin-mediated loop extrusion and A/B compartmentalization. Numerous studies have shown that loss of cohesin or reduced loop extrusion leads to stronger compartment signals, whereas increased cohesin residence or enhanced extrusion weakens compartmentalization. Given this framework, an obvious alternative explanation for the authors' observations is that the abrupt increase in compartment strength at G1/S, and its decline toward G2, could reflect cell-cycle-dependent modulation of cohesin activity rather than a compartment-intrinsic "maturation" program.

The manuscript does not explicitly consider this possibility, nor does it examine loop extrusion-related features (such as loop strength, insulation, or stripe patterns) across the same cell-cycle stages. Without discussing or analyzing this widely accepted model, it is difficult to distinguish whether the reported compartment dynamics represent a novel architectural mechanism or an indirect consequence of known changes in extrusion behavior during the cell cycle. I strongly encourage the authors to analyze their data to determine if they observe anti-correlated loop changes at the same time they observe compartment changes. Ideally, the authors would remove loop extrusion during interphase using well-established cohesin degrons available in mESCs and determine if the relative differences in compartment dynamics persist.

We thank the reviewer for raising this interesting point. We agree that there is a well-established anti-correlation between cohesin-mediated loop extrusion and A/B compartment strength in the literature.

To test whether cell cycle compartment dynamics, particularly compartment maturation at the G1/S transition, could be explained by changes in loop extrusion, we analyzed insulation at RAD21/CTCF sites (mESC data from Hansen et al., eLife, 2017) across the cell cycle. During normal cycling, we indeed observed an anti-correlation: insulation dropped as compartment strength increased at the G1/S transition. However, in G1/S-arrested cells, insulation did not drop compared to late G1 (it even slightly increased) even though compartment maturation still occurred, indicating that the two processes can be uncoupled. This is consistent with other studies showing that loop extrusion and compartment dynamics are driven by independent mechanisms (Nora et al., Cell, 2017; Zhang et al., Nat Commun, 2021), although we cannot fully rule out some contribution from loop extrusion dynamics without direct cohesin degron experiments.

We have added a new Results section describing these findings titled “Compartment maturation is independent of cohesin-mediated loop extrusion”, including new Figure panels 3H, I, and Figure S7.

(3) The proposed "peninsula-like" A-domain structures are inferred from ensemble Hi-C data and polymer modeling, rather than directly observed physical conformations. That is, single-cell imaging data clearly have shown that Hi-C (especially ensemble Hi-C) cannot uniquely specify physical conformations and that different underlying structures can produce similar contact patterns. The "peninsula" language, as written, risks being interpreted as a literal structural model rather than a conceptual visualization. Instead of risking this as just another nuanced Hi-C feature in the field, the authors could strengthen the manuscript by either (i) explicitly framing the peninsula model as a heuristic description of contact redistribution rather than a definitive physical architecture, or (ii) discussing alternative structural scenarios that could give rise to similar Hi-C patterns. Clarifying this distinction would improve the rigor and help readers better understand what aspects of A-compartment consolidation are directly supported by the data versus model-based extrapolations. For example, it would be useful to clarify whether the observed increase in long-range A-A contacts reflects spatial extension of internal A regions, changes in loop extrusion dynamics, increased compartment mixing within the A state, or population-averaged heterogeneity across alleles.

We thank the reviewer for this important clarification. We agree that the "peninsula" model should be framed as a heuristic description. As detailed in our response to Reviewer #1 (see above), we have added a disclaimer to the manuscript and provided orthogonal DNA FISH support for physical extension of A-domains during S phase. We have also ensured that the language emphasizes the conceptual nature of the model.

(4) The extension of the analysis to additional cell types using HiRES single-cell data is a valuable addition and supports the idea that compartment maturation is not unique to mESCs. However, the limitations of these data, in particular, the limited phase resolution, in addition to the pseudo-bulk aggregation and variable coverage, should be emphasized more clearly in the main text. Framing these results as evidence for conservation in principle, rather than definitive proof of identical dynamics across tissues, would be a more appropriate framing.

We agree with the reviewer. We have already explicitly acknowledged the limited temporal resolution and variable coverage of the HiRES dataset in the main text. To better reflect its supporting role, we have moved the HiRES figure (previously Fig. 4) to Fig. S10 and merged the corresponding results section with the previous one titled: “Formation of a consolidated A compartment in S-phase”.

We have also revised the language to avoid overstatement. The original conclusion read: “Together, these findings strongly indicate that compartment maturation and the accompanying A compartment consolidation represent a robust and universally observed feature across different developmental contexts.”

This has been changed to: “Together, these findings support the notion that compartment maturation and the accompanying A-compartment consolidation are not unique to mESCs and may represent a broadly conserved feature of mammalian chromatin organization.”

Similarly, the abstract has been adjusted from: “Moreover, compartment maturation was not limited to mESCs but was also observed across different developmental contexts in mice.” to: “Moreover, compartment maturation was not limited to mESCs but was also evident across different developmental contexts in mice.”

These changes frame the results as evidence for conservation in principle rather than definitive proof of identical dynamics across tissues.

**Recommendations for the authors:**

**Reviewer #1 (Recommendations for the authors):**

Please address the minor points in the public review.

In addition, on page 7, line 285: "In contrast, interactions showed minimal change across all distances though interphase". Do the authors mean "In contrast, B-B interactions..."?

We thank the reviewer for catching this. The sentence has been corrected.
